# Global research landscape of ferroptosis in gastric cancer: a multidisciplinary bibliometric analysis based on multiple databases (2017-2025)

**DOI:** 10.3389/fimmu.2025.1726253

**Published:** 2026-01-16

**Authors:** Nianshan Ji, Yiyao Cheng, Yuheng Peng, Zhiguang Sun

**Affiliations:** The First Clinical Medical College, Nanjing University of Chinese Medicine, Nanjing, China

**Keywords:** bibliometrics, ferroptosis, gastric cancer, non-coding RNAs, tumor microenvironment

## Abstract

**Objective:**

Ferroptosis, an iron-dependent regulated cell death mechanism, has recently gained attention for its crucial role in tumor progression and immune regulation. Gastric cancer (GC), one of the most common and deadly malignancies worldwide, remains a major clinical challenge. This study aims to systematically analyze the global research landscape, hotspots, and trends of ferroptosis in gastric cancer from 2017 to 2025, with a focus on its immunological implications.

**Methods:**

Publications related to ferroptosis and gastric cancer published between January 1, 2017 and April 22, 2025 were retrieved from the Web of Science Core Collection (WoSCC) and Scopus databases (n = 974). Bibliometric and visualization analyses were performed using R software (bibliometrix package), VOSviewer, and CiteSpace to explore publication trends, collaboration patterns, keyword co-occurrence, and thematic evolution.

**Results:**

Research output showed a continuous upward trend, with China leading in publication volume, followed by the United States, Japan, Canada, and South Korea. Frontiers in Pharmacology and Frontiers in Oncology were the most productive journals, while Cell and Nature were the most frequently cited. The most prominent themes included “immune checkpoint,” “tumor immune microenvironment,” “ferroptosis mechanisms,” “drug resistance,” and “non-coding RNAs.” Notably, the STAT3–ferroptosis axis and immune pathways were identified as key translational targets.

**Conclusion:**

Ferroptosis has become an important research frontier in gastric cancer and is increasingly recognized as a promising therapeutic strategy to modulate the tumor immune microenvironment and enhance immunotherapy efficacy. This study provides a comprehensive overview of the global research landscape, offering valuable guidance for future studies in cancer immunology and ferroptosis-based therapy.

## 1 Introduction

Gastric cancer is the fifth most common malignant tumor and the fourth leading cause of cancer-related death, with more than one million new cases and over 700,000 deaths annually (1, 2). In 2022, a total of 358,700 new gastric cancer cases and 260,400 related deaths were recorded in China, ranking third in mortality among all malignant tumors (3). Treatments for gastric cancer include surgical resection, chemotherapy, immunotherapy, and targeted therapy (4). Although diagnostic and therapeutic techniques have improved in recent years, gastric cancer is frequently detected at a late stage, presenting challenges such as treatment resistance and high recurrence rates. Therefore, investigating the underlying mechanisms of gastric cancer has become increasingly important. Recent developments in gastric cancer research have highlighted the importance of molecular mechanisms, such as autophagy and apoptosis, in the pathogenesis of gastric cancer (5, 6). Among these, ferroptosis has emerged as a potential therapeutic target (7).

Given this context, an increasing number of researchers have begun to focus on ferroptosis, a relatively new member of the regulated cell death family. Characterized by its reliance on iron-dependent lipid peroxidation, ferroptosis diverges markedly from classical cell death mechanisms such as apoptosis and necrosis, not only in morphological presentation but also in its underlying biochemical and genetic pathways (8). The initiation of ferroptosis involves complex signaling pathways, with the core mechanism centered on Fe²^+^- or lipoxygenase-mediated lipid peroxidation of highly expressed polyunsaturated fatty acids in cell membranes. This process interferes with cystine/glutamate antiporter systems, glutathione peroxidase activity, and polyunsaturated fatty acid overload, thereby modulating the cell’s antioxidant defense system to execute ferroptosis (9). In cancer biology, ferroptosis is believed to play a dual role in tumor suppression and promotion, offering a novel perspective for developing innovative cancer treatment strategies (10). Recent studies suggest that managing iron metabolism and promoting lipid peroxidation might help counteract chemoresistance as part of a supportive treatment approach. Researchers are currently investigating several ferroptosis inducers, such as Erastin and Tanshinone IIA, for their ability to increase the sensitivity of gastric cancer cells to chemotherapy and thereby improve overall treatment effectiveness (11). For instance, studies have shown that apatinib can induce ferroptosis in gastric cancer cells by downregulating GPX4 expression through inhibition of the transcription factor Sterol Regulatory Element-Binding Protein 1a (SREBP-1a), and that multidrug-resistant gastric cancer cells are particularly susceptible to apatinib-induced GPX4 suppression (12). In addition, miR-522 secreted by cancer-associated fibroblasts (CAFs) can inhibit ferroptosis and promote chemoresistance in gastric cancer (13). However, the current lack of analysis on research hotspots and frontiers in this field hampers researchers from accurately and rapidly identifying future research directions. Bibliometrics, as a scientific quantitative analysis tool, reveals development trends, hot topics, and frontier areas within specific research domains through statistical analysis of scientific literature (14). The bibliometric evaluation of ferroptosis and gastric cancer research not only provides a comprehensive review of the current state of research but also identifies existing gaps and potential future directions, offering valuable insights for subsequent foundational studies and clinical applications. Therefore, this study aims to apply bibliometric techniques to thoroughly examine the relationship between ferroptosis and gastric cancer, with the goal of offering useful guidance and references for researchers and clinicians in this field, thereby promoting further advancements. To date, and to the best of our knowledge, bibliometric studies systematically mapping the global research landscape of ferroptosis in gastric cancer remain limited, particularly with respect to research hotspots, mechanistic themes, thematic evolution, and immunology- or immunotherapy-related perspectives. Although individual experimental studies have explored the roles of ferroptosis in tumor biology, drug resistance, and immune regulation, the field lacks an integrated, quantitative synthesis that clarifies major contributors, emerging trends, intellectual structures, and future research directions. Therefore, this study aims to fill this gap by conducting a comprehensive bibliometric analysis of publications on ferroptosis in gastric cancer, identifying key research hotspots and collaboration patterns, evaluating temporal and thematic evolution, and highlighting potential intersections between ferroptosis and immunotherapy that may guide future investigations.

## 2 Materials and methods

### 2.1 Literature sources and retrieval strategies

The literature search was conducted on April 22, 2025, using two major databases: the Web of Science Core Collection (WoSCC) and Scopus. For both databases, the search was designed to capture studies focusing on ferroptosis and gastric cancer published between January 1, 2017 and April 22, 2025.

To ensure reproducibility, the core elements of the search strategy are summarized here. The main search terms combined ferroptosis-related keywords (e.g., “ferroptosis” OR “iron-induced cell death”) with gastric cancer-related terms (e.g., “gastric cancer”, “stomach cancer”, “gastric adenocarcinoma”).

In WoSCC, the search was performed using the Topic (TS) field, which covers title, abstract, author keywords, and Keywords Plus, with restrictions applied to document type (article or review) and language (English).

In Scopus, the equivalent search was conducted in the TITLE-ABS-KEY fields using the same conceptual keyword combinations, with limits applied to publication year, document type (article or review), and language (English).

The complete detailed search strings for both databases are provided in Annex 1.

For example, the WoSCC search was conducted using a Topic (TS) query such as TS = (“ferroptosis” OR “iron-induced cell death”) AND TS = (“gastric cancer” OR “stomach cancer” OR “gastric adenocarcinoma”), with limits applied to publication years (2017–2025), document types (article or review), and language (English).

### 2.2 Data analysis

Due to differences in data formats and structures between WoSCC and Scopus, directly merging the two datasets would likely result in information loss. To ensure data integrity, each dataset was analyzed independently. Considering the availability of structured cited-reference metadata and standardized export fields in WoSCC, the primary bibliometric analysis in this study was conducted based on WoSCC records. Results from Scopus, including annual publication trends and keyword clustering, are presented as **Supplementary Material** for reference.

Annual publication trends were analyzed using Origin 2018 software. In addition, several bibliometric and visualization tools were employed, including the bibliometrix package in R (version 4.3.3, http://www.bibliometrix.org) (15), VOSviewer (v1.6.20) (16), and CiteSpace (v6.4.R1) (17), which collectively enabled the creation of knowledge maps and the visualization of research structures. Furthermore, journal impact factors (IFs) were obtained from the 2023 Journal Citation Reports (JCR).

To ensure methodological transparency, the key analytical thresholds applied in the bibliometric analyses are summarized here. For VOSviewer (WoSCC dataset), countries with at least one publication and institutions with two or more publications were included in the co-authorship analysis. For the co-citation analysis, journals or references cited 28 times or more were retained. For keyword co-occurrence analysis, terms appearing in at least three publications were included, with “ferroptosis” and “gastric cancer” excluded due to their universal presence.

For VOSviewer (Scopus dataset), the minimum occurrence threshold for keyword co-occurrence analysis was 29 occurrences, excluding search-query terms and their synonyms.

For CiteSpace, the time slicing was set from 2017 to 2025, using a 1-year per slice interval.

The above thresholds represent the essential criteria guiding node selection and network construction, whereas the complete software configurations remain available in Annex 2.

## 3 Results

### 3.1 General overview of the documents on ferroptosis and gastric cancer

**Figure 1:**
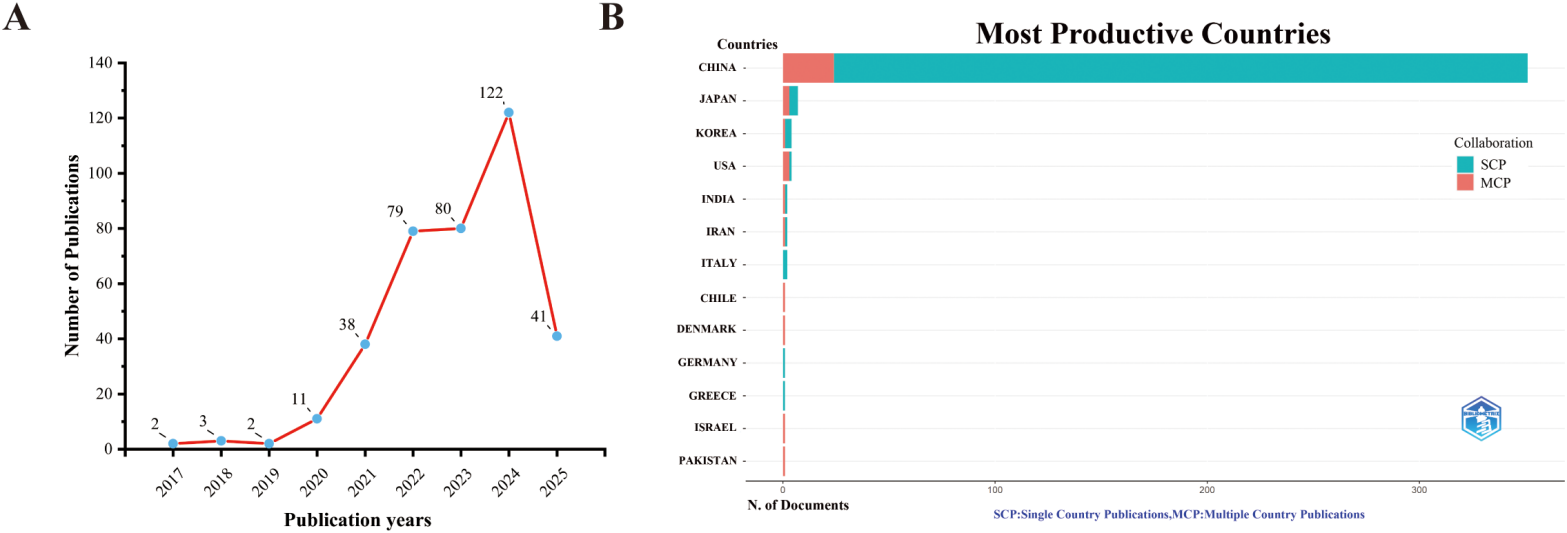
Trends in annual publication outputs the relationship between ferroptosis and gastric cancer from 2017 to 2025. **(A)** Trends of annual publication outputs. **(B)** Distribution of corresponding authors’ countries and cooperation.

A total of 378 unique records were retrieved from WoSCC after duplicates were removed. From 2017 to 2025, the number of publications on ferroptosis and gastric cancer has steadily increased, as shown in **Figure 1A**. After eliminating duplicate entries, 596 unique records were obtained from the Scopus database. The annual publication trend closely parallels that observed in WoSCC (**Supplementary Figure S1**). This upward trend reflects the growing academic interest in investigating the link between ferroptosis and gastric cancer. In total, the initial search yielded 974 documents across both databases (378 from WoSCC and 596 from Scopus); these figures represent database-specific counts rather than a cross-database de-duplicated total. Because the two databases differ in metadata structure and test merges resulted in loss of key citation information, the datasets were not merged. Accordingly, WoSCC served as the primary source for the bibliometric analysis, with Scopus findings presented as supplementary reference.

**Figure 2:**
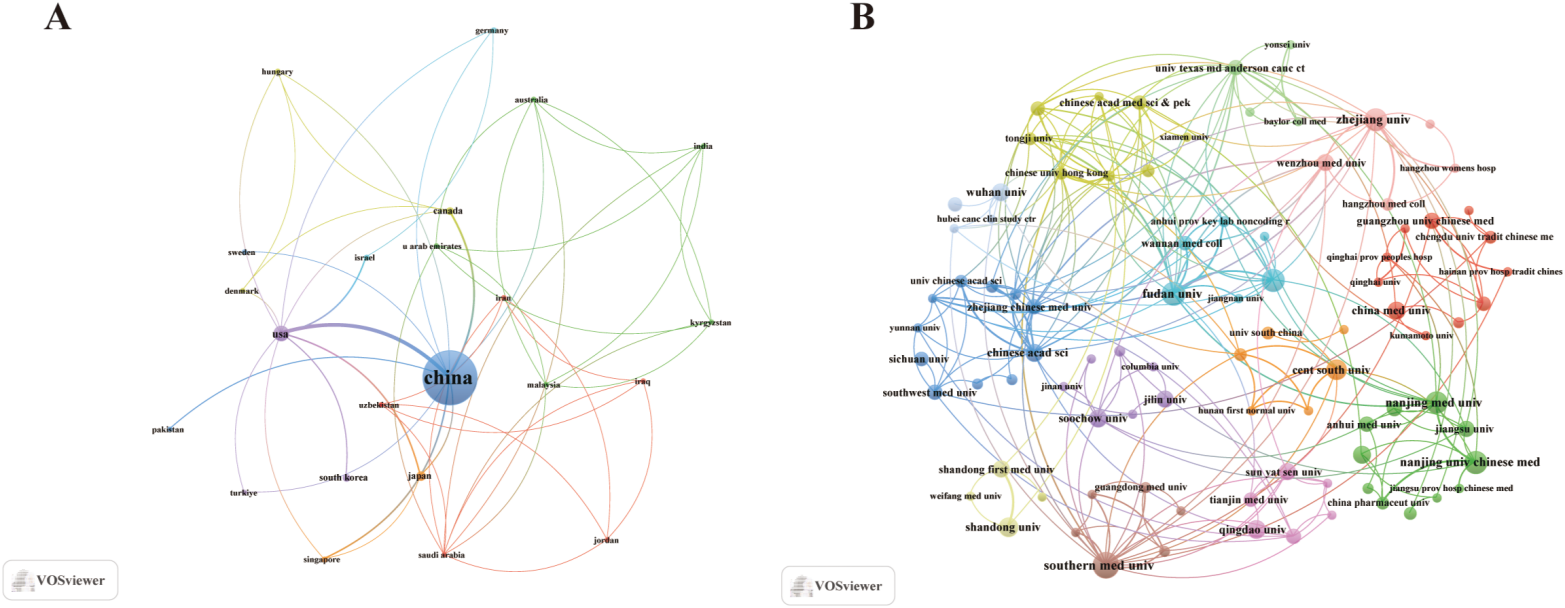
Map of countries/regions and institutions the relationship between ferroptosis and gastric cancer from 2017 to 2025. **(A)** Map of cooperation between different countries. **(B)** Map of cooperation between different institutions.

An analysis of the corresponding authors’ countries revealed that China (n = 351) led in the number of publications, followed by the Japan (n = 7), South Korea (n = 4), and USA (n = 4). Furthermore, 6.8% of China’s publications and 42.9% of Japan’s publications involved multi-country collaborations (MCPs), as shown in **Figure 1B**, **Table 1**. **Figure 2A** further highlights that China has the most extensive international collaboration in the field of ferroptosis and gastric cancer. This pattern may be associated with multiple factors, including the high disease burden of gastric cancer in China, alongside differences in research infrastructure and international collaboration capacity. Additionally, the collaboration map indicates that Southern Medical University China (n = 21) and Fudan University (n = 17) are prominent research centers for collaboration (**Figure 2B**, **Table 2**).

**Table 1:** Most relevant countries by corresponding authors of the relationship between ferroptosis and gastric cancer.

Country	Articles	SCP	MCP	Freq	MCP_Ratio
CHINA	351	327	24	0.929	0.068
JAPAN	7	4	3	0.019	0.429
KOREA	4	3	1	0.011	0.25
USA	4	1	3	0.011	0.75
INDIA	2	1	1	0.005	0.5
IRAN	2	1	1	0.005	0.5
ITALY	2	2	0	0.005	0
CHILE	1	0	1	0.003	1
DENMARK	1	0	1	0.003	1
GERMANY	1	1	0	0.003	0
GREECE	1	1	0	0.003	0
ISRAEL	1	0	1	0.003	1
PAKISTAN	1	0	1	0.003	1

SCP, Single Country Publications; MCP, Multiple Country Publications, MCP_Ratio = MCP/Articles.

**Table 2:** Most relevant affiliations of the relationship between ferroptosis and gastric cancer.

Affiliations	Articles (n)
Southern Medical University China	21
Fudan University	17
Nanjing University Of Chinese Medicine	15
Shanghai Jiao Tong University	15
Zhejiang University	15
Nanjing Medical University	14
Central South University	12
Shandong University	11
Qingdao University	10
China Medical University	9
Chinese Academy Of Sciences	9
Soochow University China	9
Wuhan University	9
Zhengzhou University	9
Jilin University	8
Shandong First Medical University Shandong Academy Of Medical Sciences	8
Sun Yat Sen University	8
Wenzhou Medical University	8
Guangzhou University Of Chinese Medicine	7
Jiangsu University	7

### 3.2 Journal analysis and visualization

To identify the journals with the highest publication and citation contributions in the field of ferroptosis and gastric cancer, we utilized the Bibliometrix package in R software. Graphical representations were generated using the ggplot2 package, and co-citation analysis was conducted using VOSviewer.

**Figure 3:**
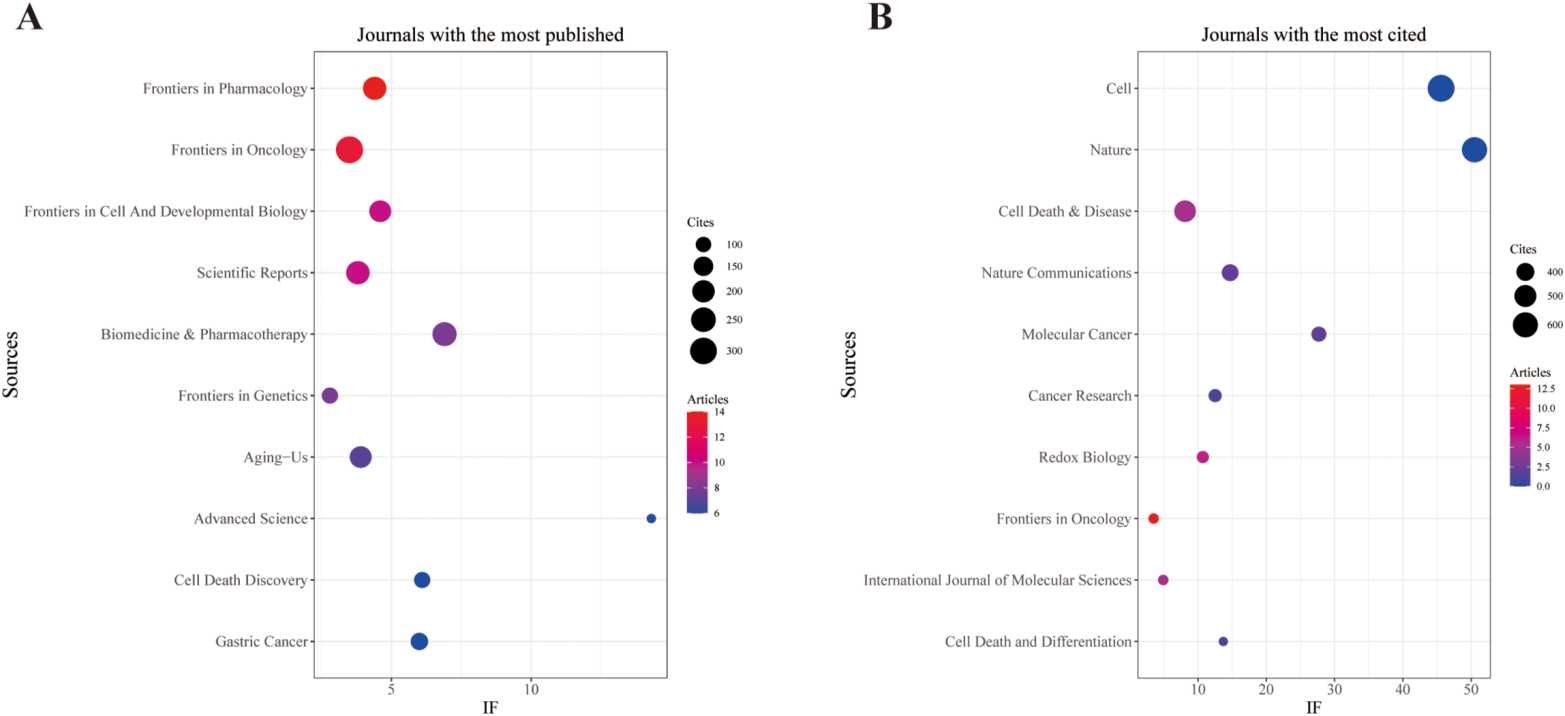
The journal with the highest volume of published articles and the journal with the most extensive citation count. **(A)** The journal with the highest quantity of published documents. **(B)** The journals with the most substantial citation counts.

**Figure 4:**
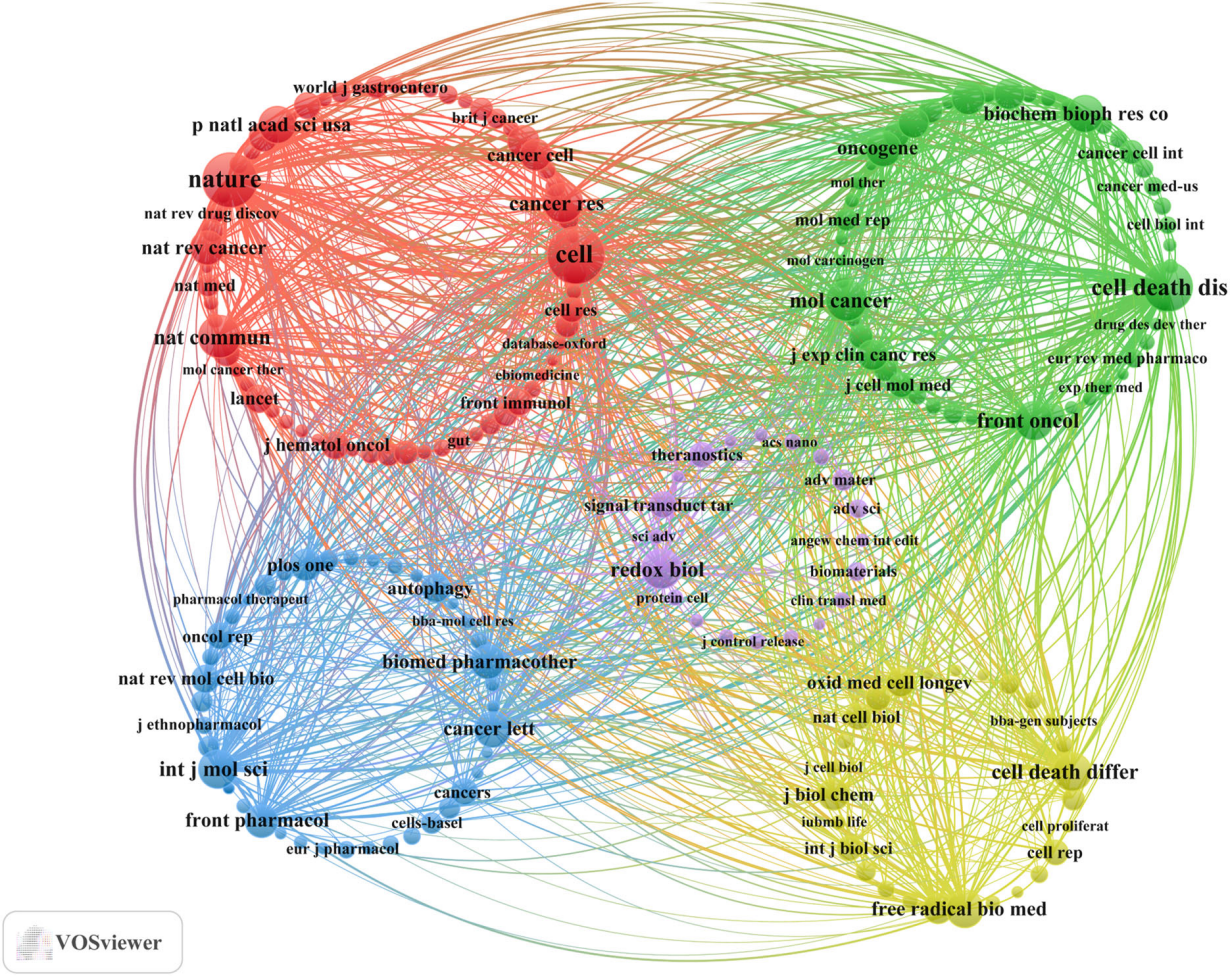
Co-cited journals involved in the relationship between ferroptosis and gastric cancer.

Our analysis resulted in a total of 378 documents published across 194 different scholarly journals (see **Supplementary Material 1** for details). As shown in **Table 3**, **Figure 3A**, Frontiers in Pharmacology (n = 14, IF = 4.4) was the leading journal, followed by Frontiers in Oncology (n = 13, IF = 3.5), Frontiers in Cell and Developmental Biology (n = 10, IF = 4.6), Scientific Reports (n = 10, IF = 3.8), and Biomedicine & Pharmacotherapy (n = 8, IF = 6.9). **Table 4**, **Figure 3B** display the most frequently cited journals, including Cell (n = 674, IF = 45.6), Nature (n = 612, IF = 50.5), Cell Death & Disease (n = 496, IF = 8.1), Nature Communications (n = 383, IF = 14.7), and Molecular Cancer (n = 355, IF = 27.7). The co-citation network in **Figure 4** indicates that Nature, Cell Death & Disease, and the International Journal of Molecular Sciences occupy central positions in the journal co-citation structure, suggesting strong intellectual linkages and shared knowledge bases in this field. These findings emphasize the significant roles of Frontiers in Oncology in ferroptosis and gastric cancer research.

**Table 3:** Top 10 journals with the most published articles.

Sources	Articles	Cites	IF (2023)
Frontiers in Pharmacology	14	225	4.4
Frontiers in Oncology	13	318	3.5
Frontiers in Cell And Developmental Biology	10	196	4.6
Scientific Reports	10	230	3.8
Biomedicine & Pharmacotherapy	8	240	6.9
Frontiers in Genetics	8	109	2.8
Aging-Us	7	192	3.9
Advanced Science	6	73	14.3
Cell Death Discovery	6	109	6.1
Gastric Cancer	6	123	6

**Table 4:** Top 10 journals with the most cited journals.

Sources	Cites	Articles	IF (2023)
Cell	674	0	45.6
Nature	612	0	50.5
Cell Death & Disease	496	5	8.1
Nature Communications	383	2	14.7
Molecular Cancer	355	2	27.7
Cancer Research	334	1	12.5
Redox Biology	327	6	10.7
Frontiers in Oncology	318	13	3.5
International Journal of Molecular Sciences	318	5	4.9
Cell Death and Differentiation	316	1	13.7

### 3.3 Citation bursts

Using the Bibliometrix package in R software, we identified the top 20 most-cited articles in the field of ferroptosis and gastric cancer, all of which were cited more than 75 times and published in 18 different academic journals (**Table 5**). This distribution indicates that influential studies are disseminated across a broad range of journals, reflecting the multidisciplinary nature of ferroptosis research in gastric cancer and the absence of a single dominant journal outlet in terms of highly cited papers. The most frequently cited articles include “CAF secreted miR-522 suppresses ferroptosis and promotes acquired chemo-resistance in gastric cancer,” “Polyunsaturated fatty acid biosynthesis pathway determines ferroptosis sensitivity in gastric cancer” and “Inhibition of STAT3-ferroptosis negative regulatory axis suppresses tumor growth and alleviates chemoresistance in gastric cancer”. These studies primarily provide comprehensive insights into the relationship between ferroptosis and gastric cancer.

**Table 5:** Top 20 cited references related to the relationship between ferroptosis and gastric cancer.

Paper	DOI	Total Citations	TC per Year
ZHANG HY, 2020, MOL CANCER	10.1186/s12943-020-01168-8	711	118.50
LEE JY, 2020, P NATL ACAD SCI USA	10.1073/pnas.2006828117	297	49.50
OUYANG SM, 2022, REDOX BIOL	10.1016/j.redox.2022.102317	241	60.25
WANG Y, 2022, CELL DEATH DIFFER	10.1038/s41418-022-01008-w	238	59.50
HAO SH, 2017, NEOPLASIA	10.1016/j.neo.2017.10.005	225	25.00
KIM EH, 2018, CANCER LETT	10.1016/j.canlet.2018.06.018	210	26.25
NIE JH, 2018, J CANCER RES CLIN	10.1007/s00432-018-2740-3	181	22.63
FU DZ, 2021, CELL MOL BIOL LETT	10.1186/s11658-021-00271-y	170	34.00
YANG H, 2022, J ADV RES	10.1016/j.jare.2021.10.001	165	41.25
GUAN ZH, 2020, BIOSCIENCE REP	10.1042/BSR20201807	161	26.83
LI DB, 2023, ONCOGENE	10.1038/s41388-022-02537-x	146	48.67
LIN ZH, 2022, REDOX BIOL	10.1016/j.redox.2022.102312	132	33.00
JIANG N, 2021, CELL DEATH DISCOV	10.1038/s41420-021-00407-1	129	25.80
NI HW, 2021, STEM CELL RES THER	10.1186/s13287-021-02394-7	120	24.00
WANG C, 2020, AGING-US	NA	105	17.50
ZHAO LY, 2021, GASTRIC CANCER	10.1007/s10120-021-01159-8	103	20.60
ZHU DM, 2022, BIOMATERIALS	10.1016/j.biomaterials.2022.121462	98	24.50
QU XL, 2023, DRUG RESIST UPDATE	10.1016/j.drup.2023.100936	84	28.00
YUAN LQ, 2020, AGING-US	10.18632/aging.102836	80	13.33
GAO ZW, 2020, BIOMED PHARMACOTHER	10.1016/j.biopha.2020.110092	79	13.17

**Figure 5:**
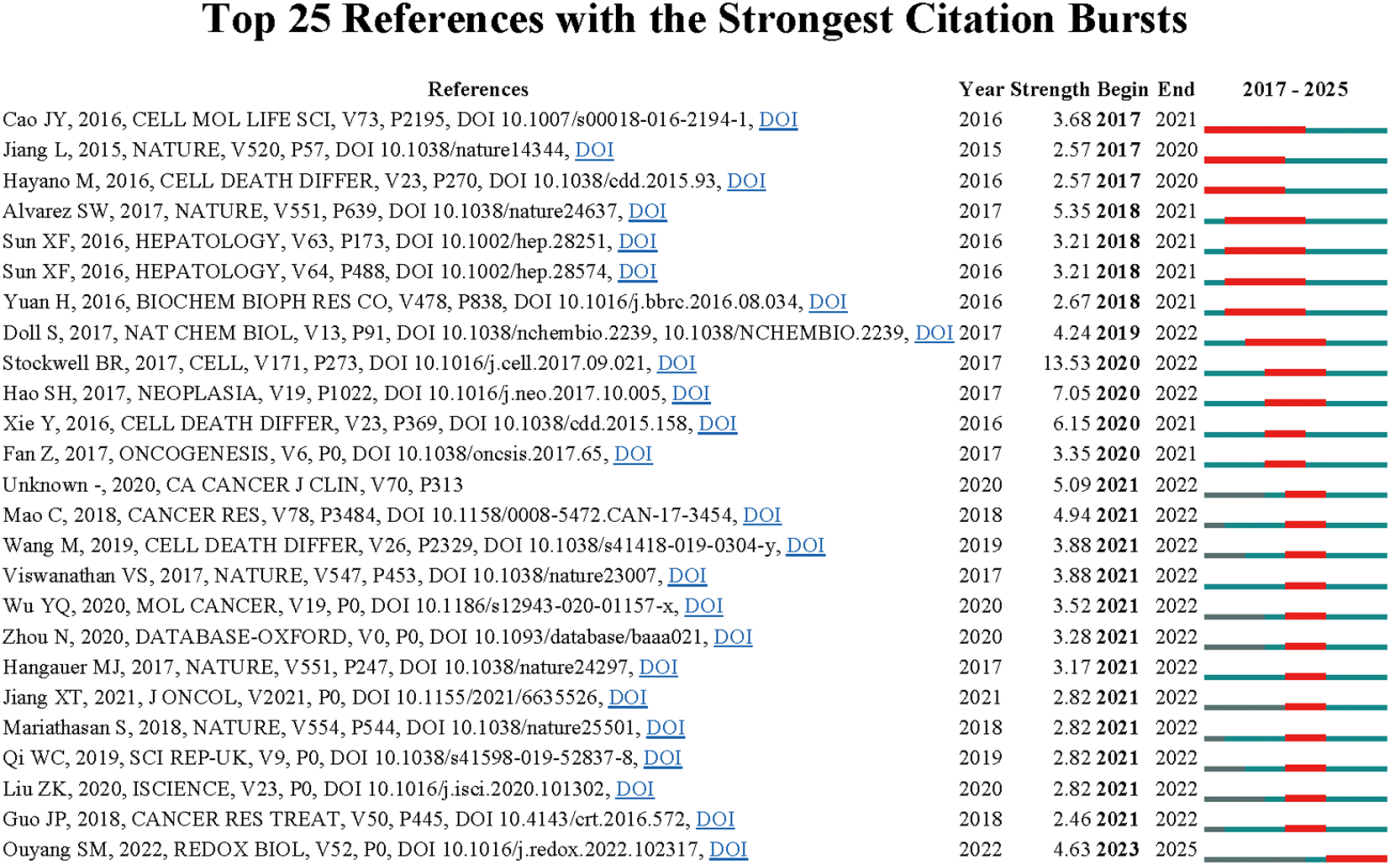
Top 25 references with the strongest citation bursts on the relationship between ferroptosis and gastric cancer research.

Additionally, to identify citation bursts in the context of ferroptosis and gastric cancer, we used CiteSpace to detect 30 references with notable citation bursts, based on specific criteria (top 25; status count: 2; minimum duration: 2). Of these, 25 references are displayed in **Figure 5**, with their titles and DOIs listed in **Supplementary Material 2**. The top three references exhibiting the most pronounced citation bursts were: (1) “Ferroptosis: A Controlled Cell Death Mechanism Connecting Metabolic Pathways, Oxidative Homeostasis, and Disease (strength: 13.53);” (2) “Cysteine Dioxygenase 1 Functions as a Key Modulator of Erastin-Induced Ferroptosis in Gastric Cancer Cells (strength: 7.05);”(3) “Ferroptosis: process and function (strength: 6.15).” Additionally, the most cutting-edge citations included: (1) “Inhibition of STAT3-ferroptosis negative regulatory axis suppresses tumor growth and alleviates chemoresistance in gastric cancer;” (2) “Ferroptosis: A Novel Anti-tumor Action for Cisplatin;” (3) “Systematic Analysis of the Aberrances and Functional Implications of Ferroptosis in Cancer”.

Through our analysis of the most-cited references and citation bursts, we identified two key areas of focus within the field of ferroptosis in gastric cancer: 1. Investigating the role of ferroptosis in chemotherapy resistance in gastric cancer cells, and strategies to overcome resistance by inducing ferroptosis.; 2. Exploring key molecular regulators and their mechanisms in modulating ferroptosis within gastric cancer contexts.

### 3.4 Keyword clusters and evolution of themes

Keyword clusters are essential for understanding the primary research themes and directions in any given field. In this study, VOSviewer was employed to analyze 1,470 keywords. **Table 6** displays the top 20 most frequent keywords, each appearing more than 21 times, representing prominent research focuses. The most common keyword was “cell” (n = 65), followed by “apoptosis” (n= 56), “expression” (n= 47), “mechanism” (n = 45), “proliferation” (n = 38), and “autophagy” (n = 36).

**Table 6:** Top 20 keywords related to the relationship between ferroptosis and gastric cancer.

Rank	Keywords	Count
1	cell	65
2	apoptosis	56
3	expression	47
4	mechanism	45
5	proliferation	38
6	autophagy	36
7	activation	35
8	pathway	32
9	resistance	32
10	death	31
11	oxidative stress	31
12	prognosis	30
13	metabolism	29
14	progression	28
15	gpx4	27
16	growth	26
17	lncrna	23
18	ros	23
19	lipid peroxidation	22
20	colorectal cancer	21

**Figure 6:**
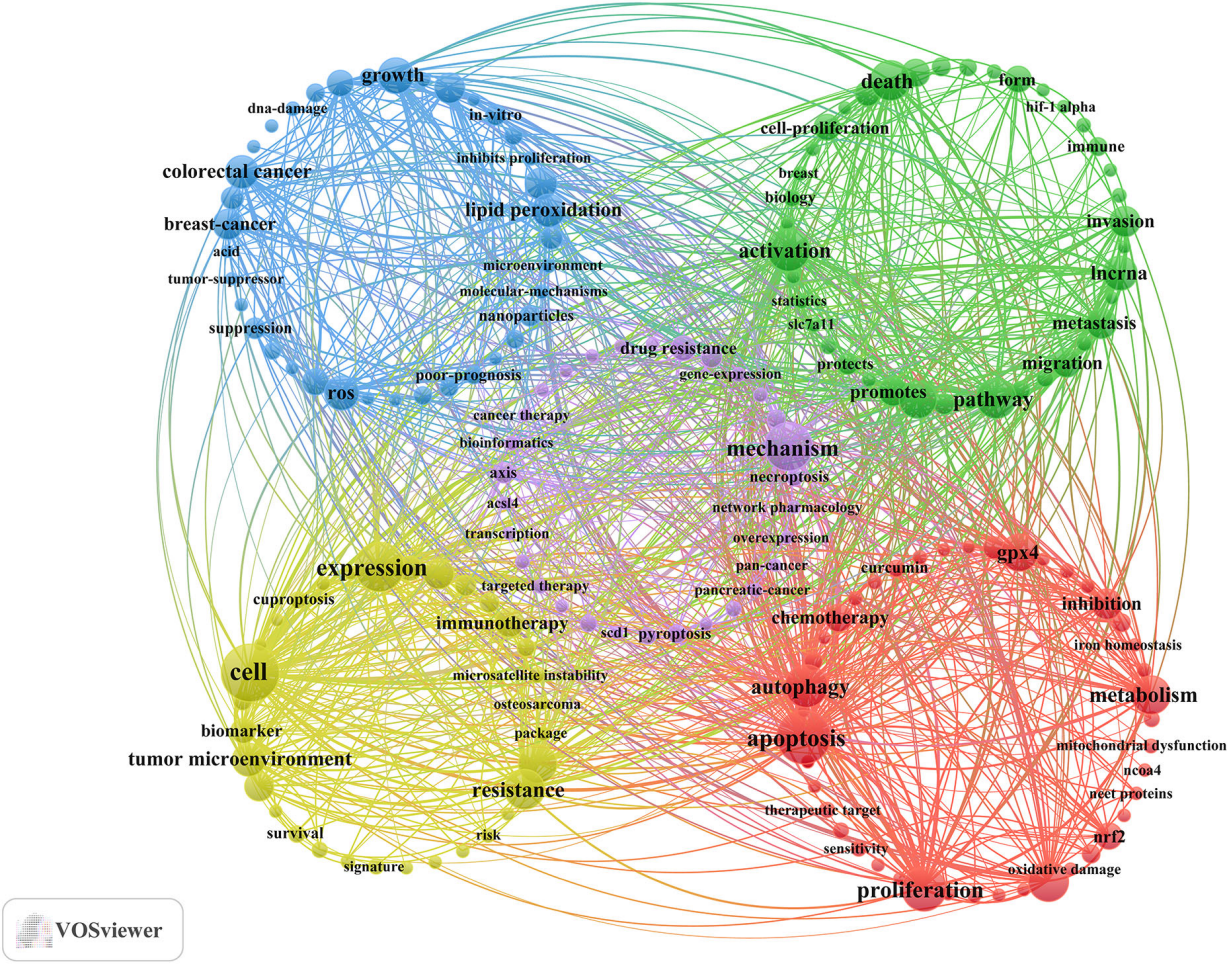
Keyword co-occurrence map of publications on the relationship between ferroptosis and gastric cancer.

Cluster analysis revealed five distinct clusters, shown in **Figure 6**: (1)The intersection of ferroptosis, oxidative stress, and various cell death mechanisms in gastric cancer research (red dots), there are 43 keywords, including apoptosis, proliferation, autophagy, oxidative stress, metabolism, and so on. (2) The role of ferroptosis-related signaling pathways and their multilayered regulatory networks in the progression and invasion of gastric cancer (green dots), there are 40 keywords, including activation, pathway, death, progression, lncrna, and so on. (3) Mechanistic insights into ROS-mediated(Reactive Oxygen Species-mediated) lipid peroxidation in regulating ferroptosis and its implications in gastric cancer (blue dots), there are 37 keywords, including growth, ros, lipid peroxidation, colorectal cancer, iron, and so on. (4) Integrative analysis of ferroptosis-related gene expression, tumor immune microenvironment, and emerging cell death modalities for prognostic model construction in gastric cancer (yellow dot), there are 32 keywords, including cell, expression, resistance, prognosis, tumor microenvironment, and so on. (5) Bioinformatics- and network pharmacology-based exploration of lipid metabolism and multiple cell death mechanisms in gastric cancer drug resistance and tumor progression (purple dot), there are 30 keywords, including mechanism, drug resistance, axis, epidemiology, lipid metabolism, and so on. All keywords in the five clusters can be found in **Supplementary Material 3**. In addition, VOSviewer was employed to identify 169 keywords from Scopus (**Supplementary Figure S2**). Current research on ferroptosis in gastric cancer converges on several key hotspots: (1) the molecular mechanisms and signaling pathways of ferroptosis in cancer development, drug response, drug resistance, and immune regulation, including gastric cancer; (2) experimental studies of ferroptosis in gastric cancer using cell lines and animal models, with a focus on cellular and molecular mechanisms as well as pharmacological effects; and (3) the application of ferroptosis-related biomarkers and gene expression analyses in clinical studies, encompassing prognosis prediction, immunotherapy response, and translational research in gastric cancer patients.

**Figure 7:**
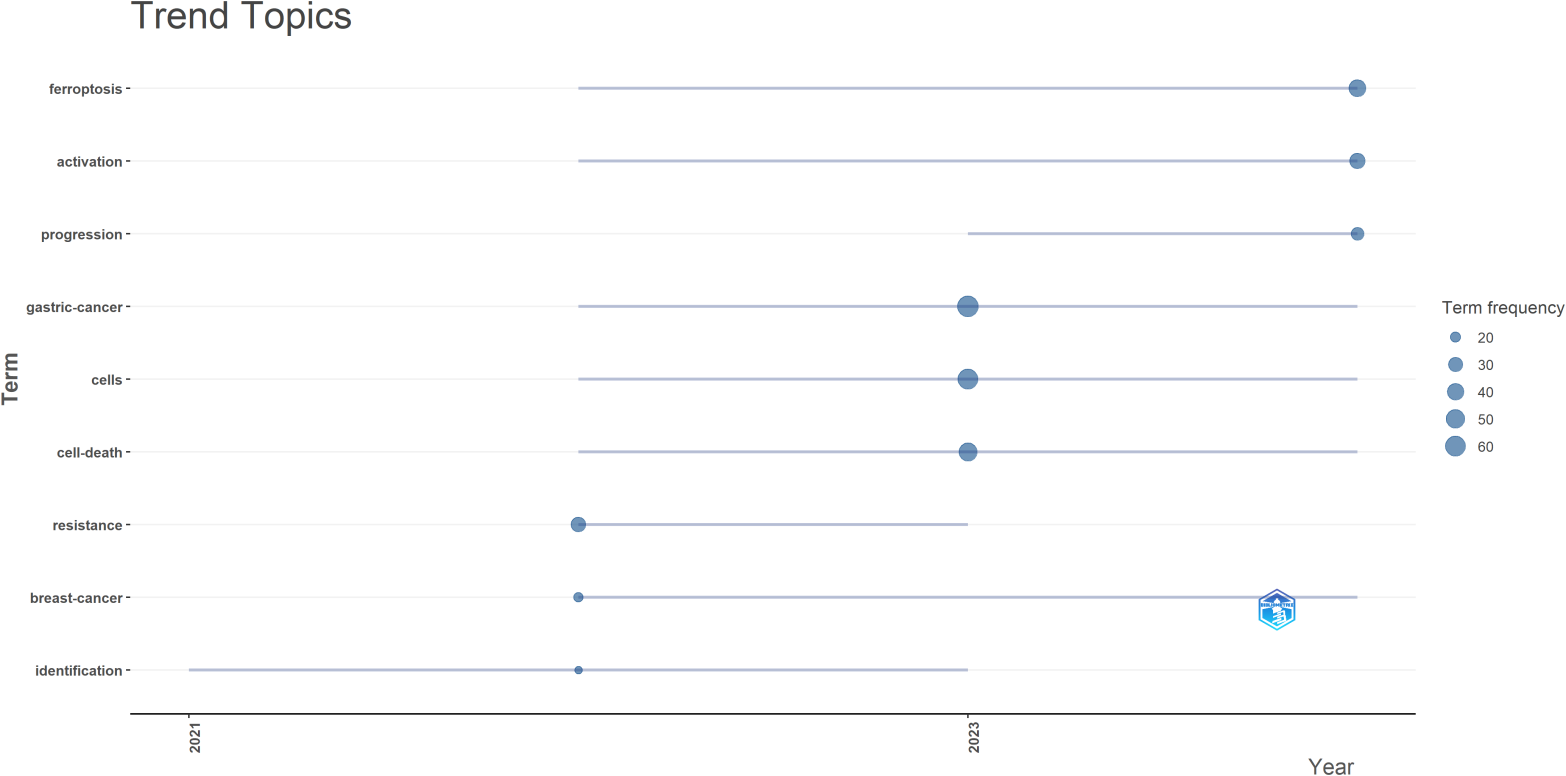
Trend topics on the relationship between ferroptosis and gastric cancer.

Furthermore, to project future trends in this domain, we used the bibliometrix toolkit in the R programming environment to create a dynamic thematic progression chart (**Figure 7**). Since the initial identification of ferroptosis-related molecular markers in 2021, research rapidly shifted in 2022–2023 toward elucidating the specific mechanisms of ferroptosis and resistance-escape pathways in gastric cancer cell models. By 2023-2024, the field had entered a translational phase, focusing on strategies to activate ferroptosis and evaluate its impact on tumor progression. Future studies are expected to focus on integrated multimodal combination therapies, the development of clinical biomarkers reflecting ferroptosis sensitivity, and precise interventions targeting ferroptosis-related interactions within the tumor microenvironment. These keyword clusters and citation burst patterns collectively informed the thematic structure of our in-depth discussion in Section 4.2, where the major hotspots-drug resistance, signaling pathways, and prognostic applications-are directly interpreted in the context of the clusters identified in **Figure 6**.

### 3.5 Refined analysis of research hotspots

In summary, through a comprehensive integration of citation burst detection, keyword co-occurrence analysis, clustering, and thematic evolution, we have delineated the emerging focal points at the intersection of ferroptosis and gastric cancer. Our results indicate that the primary research priorities in this area revolve around three major aspects: (1) Regulation of Ferroptosis in Gastric Cancer Drug Resistance: Mechanisms for Reversing Chemoresistance and Enhancing Therapeutic Sensitivity(2) The Role of Ferroptosis Signaling Pathways in Gastric Cancer: A Regulatory Network of Tumor Progression, Invasion and Metastasis, and Immune Evasion. (3) Application of Ferroptosis in Precision Therapy for Gastric Cancer: Prognostic Modeling and Biomarker Development.

## 4 Discussion

### 4.1 General overview

This study compiled a curated dataset of 378 articles published between 2017 and 2025. By analyzing the trends and patterns in these publications, we found that the number of studies on the relationship between ferroptosis and gastric cancer has steadily increased from 2017 to 2025. This growth indicates a rising academic interest in this area. In the realm of ferroptosis and gastric cancer research, China has emerged as a leading country, with a significantly higher number of publications. This trend may be influenced by multiple factors, including the high incidence and mortality of gastric cancer in China, along with strong research capacity, sustained funding support, and national research priorities in oncology and biomedical research. As a result, China has been an active contributor to studies exploring ferroptosis in gastric cancer.

Of the 194 journals publishing these articles, key contributors include Frontiers in Pharmacology, Frontiers in Oncology, Frontiers in Cell and Developmental Biology, and Scientific Reports. Notably, Frontiers in Oncology have not only contributed a considerable volume of relevant literature but have also received extensive citations, establishing their status as leading journals in this field.

### 4.2 Hotspots and development trends

The thematic hotspots discussed below are directly informed by the bibliometric findings, particularly the five keyword clusters identified in **Figure 6** and the citation burst analysis. These structures reveal how the field has evolved from mechanistic studies to translational applications.

Moreover, the bibliometric clusters also highlight the growing emphasis on ferroptosis-mediated immune regulation, including immune checkpoint activity and remodeling of the tumor immune microenvironment.

#### 4.2.1 Regulation of ferroptosis in gastric cancer drug resistance: mechanisms for reversing chemoresistance and enhancing therapeutic sensitivity

This hotspot corresponds mainly to the “oxidative stress-cell death mechanism” cluster (Cluster 1, red) and the “drug resistance-lipid metabolism” cluster (Cluster 5, purple) identified in **Figure 6**, and several of the strongest citation bursts (e.g., miR-522, CDO1, AGPS) also highlight drug resistance as an emerging frontier.

Rather than reiterating its fundamental biological features, recent work has emphasized the role of ferroptosis in mediating chemoresistance and influencing therapeutic outcomes. Our literature analysis indicates that inducing or inhibiting ferroptosis pathways can reverse resistance to chemotherapeutic agents (e.g., 5-fluorouracil, platinum-based drugs) and targeted therapies (e.g., apatinib), thereby enhancing treatment sensitivity.

For example, apatinib-resistant gastric cancer cells have been shown to exhibit resistance to ferroptosis, which correlates with decreased levels of polyunsaturated ether phospholipids. This resistance is mediated by the downregulation of alkylglycerone phosphate synthase (AGPS), a key enzyme regulated by the transcription factor ELK1. Inhibition of AGPS expression has been found to restore apatinib-induced ferroptosis, thereby reversing drug resistance and providing a promising avenue for combinatory therapeutic strategies (18).

Ferroptosis inducers such as erastin and RSL3 have also demonstrated great potential in reversing chemoresistance. Erastin induces ferroptosis in gastric cancer cells by targeting CDO1.Silencing CDO1 inhibits erastin-induced ferroptosis, maintains intracellular glutathione (GSH) levels, and reduces the production of the lipid peroxidation product malondialdehyde, indicating a critical role of CDO1 in ferroptosis regulation and chemosensitivity (19).In addition, TPM4 functions as an oncogene in gastric cancer by maintaining SCD1 levels to suppress ferroptosis; knockout of TPM4 reduces SCD1 expression, alters the SFA/MUFA ratio, triggers endoplasmic reticulum stress and the unfolded protein response (UPR), eventually inducing both apoptosis and ferroptosis (20).

Natural compounds have also shown promise in promoting ferroptosis. Crocin, for instance, induces ferroptosis in gastric cancer cells via the Nrf2/GGTLC2 signaling pathway, thereby inhibiting cellular proliferation and migration, and supporting its potential as a therapeutic agent against drug resistance (21). Dihydroartemisinin acts synergistically with cisplatin to disrupt antioxidant defenses through the inhibition of GPX4,inducing ferroptosis and reversing chemoresistance in both *in vitro* and *in vivo* gastric cancer models (22).

In addition, ferroptosis-induced lipid peroxidation may enhance antitumor immunity by promoting antigen release and facilitating CD8^+^ T-cell-mediated cytotoxic activity, providing further rationale for overcoming chemoresistance.

Integrating ferroptosis-based strategies into clinical practice holds great promise for the management of gastric cancer (23). However, challenges remain, including the specificity and safety of ferroptosis inducers, as well as the need to elucidate all mechanisms of drug resistance. Future research should focus on optimizing combination therapies and conducting clinical trials to validate these preclinical findings.

#### 4.2.2 The role of ferroptosis signaling pathways in gastric cancer: a regulatory network of tumor progression, invasion and metastasis, and immune evasion

This theme is strongly aligned with the “signaling pathways and multilayered regulatory networks” cluster (Cluster 2, green) and the “ROS-lipid peroxidation mechanisms” cluster (Cluster 3, blue) shown in **Figure 6**, demonstrating the bibliometric basis for the mechanistic emphasis of this section. Citation bursts involving GPX4, STAT3, and SCD1 further reinforce the centrality of these pathways.

In the context of tumor biology, emerging evidence now centers on how ferroptosis contributes to malignant progression, metastatic potential, and modulation of the immune microenvironment. Aberrant expression of ferroptosis-related genes is frequently associated with accelerated tumor growth.

For instance, Tanshinone IIA induces ferroptosis by upregulating p53 and downregulating SLC7A11, thereby inhibiting gastric cancer cell proliferation and promoting cell death (24). This process involves elevated lipid peroxidation and diminished glutathione levels, suggesting that ferroptosis inducers may serve as effective therapeutic agents. Moreover, knockdown of SCD1 increases the sensitivity of gastric cancer cells to ferroptosis and suppresses tumor growth, indicating that SCD1 is a promising therapeutic target (25).

Ferroptosis signaling pathways are also critically involved in the invasion and metastasis of gastric cancer. Inducing ferroptosis markedly reduces the migratory and invasive capabilities of gastric cancer cells. For example, the traditional Chinese medicine Actinidia chinensis Planch induces ferroptosis by inhibiting GPX4 and xCT, thereby diminishing the invasiveness of gastric cancer cells (26). Additionally, cancer-associated fibroblasts (CAFs) contribute to chemoresistance and metastasis by secreting exosomal miR-522, which inhibits ferroptosis. This finding suggests that ferroptosis regulation may influence the tumor microenvironment and affect metastatic progression (13).

Ferroptosis further impacts immune evasion by modulating the tumor immune microenvironment. Ferroptosis-related gene signatures have been identified as potential biomarkers for predicting responses to immune checkpoint inhibitors, with patients exhibiting lower ferroptosis activity demonstrating improved immunotherapeutic outcomes (27). Furthermore, ferroptosis-associated long non-coding RNAs (lncRNAs) may downregulate hub ferroptosis-related genes (hub-FRGs), inhibit CD4^+^ T cell activation, and promote immune evasion, underscoring the multifaceted role of ferroptosis in immune regulation (28).

At the same time, ferroptosis-related pathways can reshape immune infiltration patterns, influence immune checkpoint expression, and modulate immunosuppressive cell subsets, further linking ferroptosis to tumor immune escape mechanisms.

Overall, the induction of ferroptosis holds considerable promise for enhancing antitumor immune responses and improving therapeutic efficacy; however, its precise molecular mechanisms warrant further investigation.

#### 4.2.3 Application of ferroptosis in precision therapy for gastric cancer: prognostic modeling and biomarker development

This hotspot corresponds directly to the “immune microenvironment-prognostic model” cluster (Cluster 4, yellow) identified in **Figure 6**, which includes high-frequency terms such as “prognosis”, “tumor microenvironment”, and “resistance”. Citation bursts related to ferroptosis-associated prognostic signatures further support this translation-oriented direction.

The integrated application of ferroptosis in the precision treatment of gastric cancer shows considerable promise, particularly in prognostic modeling and the development of clinical biomarkers.

Bioinformatics analyses of The Cancer Genome Atlas(TCGA) and Gene Expression Omnibus(GEO) databases have led to the construction of prognostic models based on ferroptosis-related genes (FRGs), enabling patient risk stratification and therapeutic guidance. For instance, researchers have developed a prognostic index (FRGPI) based on four FRGs—NOX4, MTCH1, GABARAPL2, and SLC2A3—using LASSO-Cox regression analysis to classify patients into high- and low-risk groups. Patients in the high-risk group exhibited worse overall survival, and the model correlated with disease progression and immune checkpoint protein expression. These models support personalized therapy by evaluating immune cell infiltration and drug sensitivity, with validation results indicating high predictive accuracy AUC(Area Under the Curve): 0.623–0.787 in TCGA and 0.754–0.787 in GEO) (29).

Ferroptosis-related genes and non-coding RNAs also represent promising foundations for the development of clinical biomarkers used in diagnosis, prognostic evaluation, and treatment monitoring. lncRNAs, such as NEAT1 and LINC01606, have been implicated in the regulation of ferroptosis, suggesting their potential utility in personalized cancer interventions (30). SCD1 regulates ferroptosis in gastric cancer stem cells via the SQLE/cholesterol/mTOR pathway and is associated with poor prognosis, underscoring its value as both a biomarker and therapeutic target (31). Additionally, the circular RNA circRHOT1 promotes tumor growth and suppresses ferroptosis by recruiting the acetyltransferase KAT5(Lysine acetyltransferase 5) to the GPX4 promoter, thereby upregulating GPX4 expression. This emphasizes its potential as a non-invasive biomarker (32).

Notably, several ferroptosis-related signatures correlate strongly with immune infiltration patterns-particularly CD8^+^ T-cell abundance and checkpoint expression-suggesting their potential value in guiding immunotherapy selection.

Ferroptosis holds potential in overcoming chemotherapy resistance and enhancing immunotherapy. Curcumin, for example, can activate ferroptosis by inhibiting the PI3K/AKT/mTOR signaling axis, leading to notable suppression of gastric cancer cell proliferation, and represents a promising natural candidate for ferroptosis-based precision treatment (33). Simvastatin enhances anti-PD-1 therapy by inducing ferroptosis and stimulating antitumor immunity, particularly through the activation of CD8^+^ T cells. This strategy is especially effective in microsatellite-stable gastric cancer, suggesting new avenues for immune-based precision treatment (34). Moreover, risk scoring models constructed from ferroptosis-related genes can predict patient responses to immune checkpoint inhibitors. Patients with higher risk scores tend to show more favorable immune responses and improved survival, revealing the value of these models in immuno-precision oncology (35).

### 4.3 Limitations

This research drew on data from the WoSCC database to conduct a structured bibliometric investigation, capturing the thematic distribution, influential topics, and developmental trajectories within the field. This methodology supports refined domain analysis and helps delineate directions for future inquiry. Despite its strengths, several limitations merit attention. First, while WoSCC is recognized as a trusted and widely used bibliometric data source, depending solely on this repository may lead to the exclusion of relevant literature indexed elsewhere (36–38). Second, the restriction to English-language documents could introduce a bias that limits the universality of the results. It should also be noted that visualization tools like CiteSpace and VOSviewer, though useful for mapping academic networks and topic clusters, are not a substitute for comprehensive systematic reviews, nor can they appraise the methodological robustness of individual publications. Moreover, citation metrics are influenced by time lag, meaning recently published studies may be underrepresented in citation-based assessments (39).

In addition to these general limitations, this study has several methodological constraints specific to its design. First, the search strategy required the explicit presence of the term “ferroptosis” in titles, abstracts, or author keywords. As a result, earlier studies related to iron-dependent lipid peroxidation or oxidative cell death-conducted before “ferroptosis” was formally introduced as a descriptor-may not have been captured, potentially overlooking foundational scientific contributions.

Second, the analysis did not stratify publications by study type (e.g., mechanistic, translational, or clinical research). The absence of such stratification may obscure differences in how ferroptosis-related concepts are interpreted, applied, and advanced across different stages of the research continuum.

Despite these constraints, the conclusions drawn from this analysis remain well-founded and contribute meaningful reference points for ongoing and future research in the domain (40).

## 5 Conclusion

This study provides a systematic bibliometric mapping of research trends and knowledge frontiers in the field of ferroptosis and gastric cancer. Key conclusions include:

- The ferroptosis–gastric cancer relationship has garnered increasing global attention. Countries such as China, the USA, Japan, and Canada have shown high engagement and have established active collaborative networks.
- Within this field, Frontiers in Pharmacology and Frontiers in Oncology have established themselves as key publication platforms. Additionally, journals such as Cell are frequently cited, while Frontiers in Oncology distinguish themselves through both prolific output and significant citation frequency, reinforcing their central academic standing.
- Investigating the role of ferroptosis in chemotherapy resistance in gastric cancer cells, and strategies to overcome resistance by inducing ferroptosis is among the current research hotspots.
- Current mechanistic investigations on ferroptosis in gastric cancer emphasize themes like gene regulation, lipid peroxidation dynamics, and modulation of the tumor microenvironment, including effects on CD8^+^ T-cell infiltration, immune checkpoint expression, and the composition of immunosuppressive cell subsets. The increasing relevance of non-coding RNAs and immune checkpoint pathways is also shaping novel therapeutic frameworks.
- Applying ferroptosis-related insights to develop personalized treatment modalities for gastric cancer and to optimize immunotherapy strategies—particularly by leveraging ferroptosis-related biomarkers and gene signatures to guide immune checkpoint inhibitor selection and response prediction—represents an emerging and influential direction in precision oncology research.

In conclusion, this study offers comprehensive insights into emerging research dynamics and core topics related to the ferroptosis and gastric cancer. These results not only equip researchers with essential contextual understanding but also serve as a foundation for identifying novel avenues of investigation. By outlining the present frontiers and pinpointing critical thematic areas, our analysis contributes meaningful direction and robust support for advancing future scholarly innovation, while also providing conceptual support for integrating ferroptosis-associated molecular features and immune infiltration patterns into future immunotherapy design for gastric cancer.

## Data Availability

Publicly available datasets were analyzed in this study. This data can be found here: Web of Science database and Scopus database: Direct link: https://www.webofscience.com; https://www.scopus.com.
