# Supplementary figures and images for: Global research landscape of ferroptosis in gastric cancer: a multidisciplinary bibliometric analysis based on multiple databases (2017-2025)
Source: Front Immunol. 2026 Jan 16;16:1726253. doi: 10.3389/fimmu.2025.1726253 (PMC12855500; doi:10.3389/fimmu.2025.1726253)

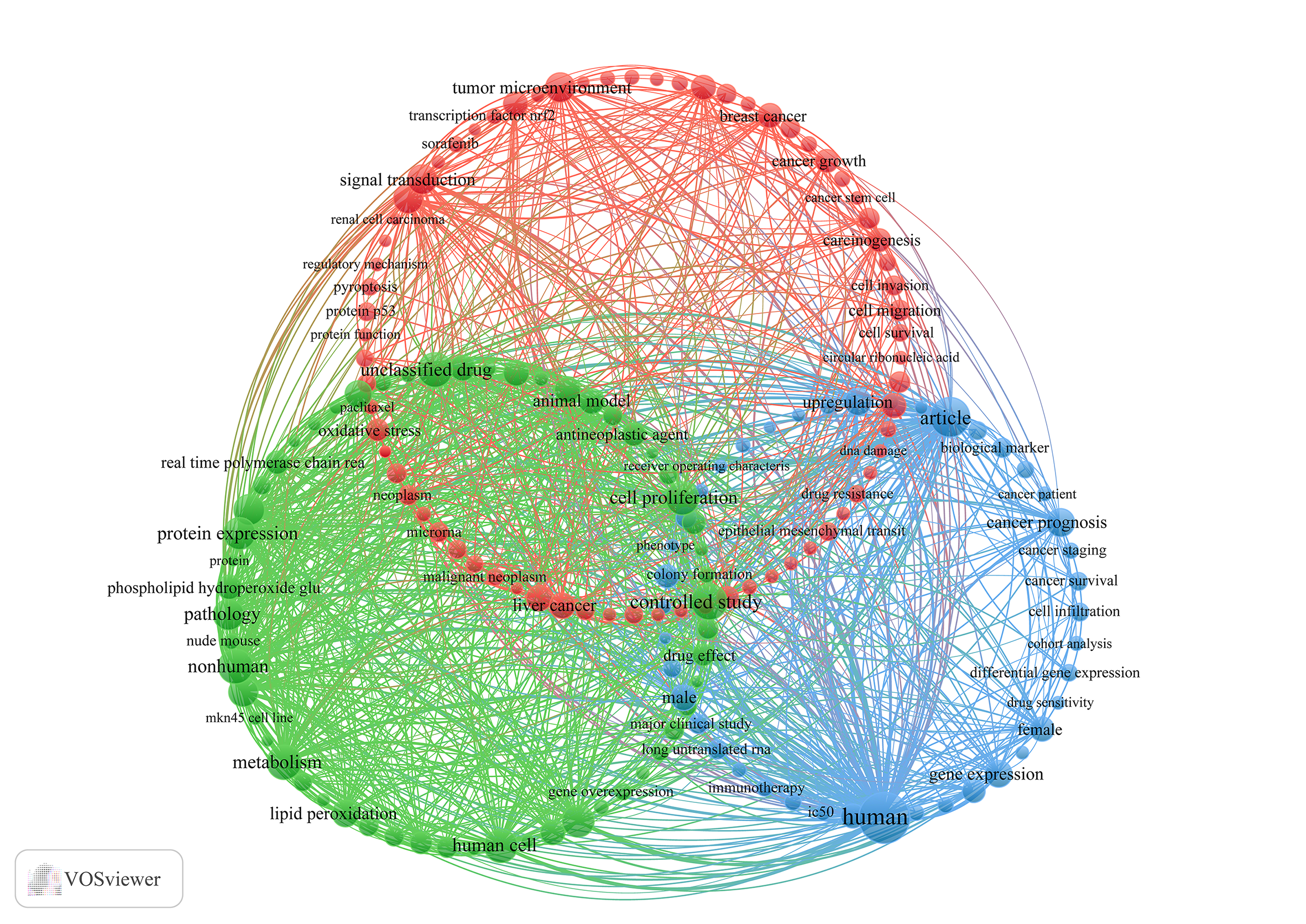

Supplement: Supplementary file 2 [file Image2.tif]
