# Supplementary material for: Global research landscape of ferroptosis in gastric cancer: a multidisciplinary bibliometric analysis based on multiple databases (2017-2025)
Source: Front Immunol. 2026 Jan 16;16:1726253. doi: 10.3389/fimmu.2025.1726253 (PMC12855500; doi:10.3389/fimmu.2025.1726253)
Supplement: Supplementary file 3 [file Table1.docx]

**Web of Science database search formula**

((((TS=(“Iron-induced cell death” OR ferroptosis)) AND TS=(“Stomach Neoplasm*” OR “Gastric Neoplasm*” OR “Cancer of Stomach” OR “Stomach Cancer*” OR “Cancer of the Stomach” OR “Gastric Cancer*” OR “Gastric Cancer, Familial Diffuse” OR “gastric adenocarcinoma” OR “gastric tumor*” OR “stomach adenocarcinoma” OR “stomach tumor*” OR “tumor of stomach”)) AND DOP=(2017-01-01/2025-04-22)) AND DT=(Article OR Review)) AND LA=(English).

**Scopus database search formula**

TITLE-ABS-KEY ( "Iron-induced cell death" OR ferroptosis ) AND TITLE-ABS-KEY ( "Stomach Neoplasm*" OR "Gastric Neoplasm*" OR "Cancer of Stomach" OR "Stomach Cancer*" OR "Cancer of the Stomach" OR "Gastric Cancer*" OR "Gastric Cancer, Familial Diffuse" OR "gastric adenocarcinoma" OR "gastric tumor*" OR "stomach adenocarcinoma" OR "stomach tumor*" OR "tumor of stomach" ) AND PUBYEAR > 2016 AND PUBYEAR < 2026 AND ( LIMIT-TO ( DOCTYPE , "ar" ) OR LIMIT-TO ( DOCTYPE , "re" ) ) AND ( LIMIT-TO ( LANGUAGE , "English" ) )
