# Supplementary material for: Global research landscape of ferroptosis in gastric cancer: a multidisciplinary bibliometric analysis based on multiple databases (2017-2025)
Source: Front Immunol. 2026 Jan 16;16:1726253. doi: 10.3389/fimmu.2025.1726253 (PMC12855500; doi:10.3389/fimmu.2025.1726253)
Supplement: Supplementary file 4 [file Table2.docx]

Vosviewer parameters (**WoSCC database**)

The inclusion criteria were as follows: countries with at least one publication and institutions with a minimum of two publications were incorporated into the co-authorship analysis. For the co-citation network, only sources cited 28 times or more were included. In the keyword co-occurrence analysis, terms that appeared in three or more articles were considered, with the keywords "ferroptosis" and "gastric cancer" deliberately excluded.

Vosviewer parameters (**Scopus database**)

for co-occurrence keyword analysis, a keyword needed a minimum of 29 occurrences, excluding keywords terms used in the search query as well as their synonyms
